# Workplace environment around physicians’ burnout: A qualitative study in French hospitals

**DOI:** 10.5271/sjweh.3977

**Published:** 2021-09-30

**Authors:** Jordan Sibeoni, Laura Bellon-Champel, Laurence Verneuil, Caroline Siaugues, Anne Revah-Levy, Olivier Farges

**Affiliations:** Service Universitaire de Psychiatrie de l’Adolescent, Argenteuil Hospital Centre, Argenteuil, France; ECSTRRA Team, UMR-1153, Inserm, Université de Paris, F-75010 Paris, France; Université de Paris, UPNVS, Paris, France; Service de chirurgie hépato-bilio-pancréatique, AP-HP, Hôpital Beaujon, F-92118 Paris, France

**Keywords:** doctor, France, mental health, occupational burnout, public hospital, qualitative research, resident, work-related issue

## Abstract

**Objective:**

Burnout among physicians in public hospital has become a major public health issue in most Western countries. Qualitative literature has underlined the importance of interpersonal and group aspects in this context. Yet, no qualitative study has ever explicitly explored workplace direct environment’s association with physicians’ burnout. This study aimed to fill this gap.

**Methods:**

This qualitative study used the five-stage inductive process to analyse the structure of lived experience (IPSE) approach and was conducted in French hospitals. We interviewed 45 participants – 16 with a lived experience of burnout and 29 of their colleagues – 19 women/26 men, (13 radiologists, 12 gastroenterologists, 10 gastrointestinal surgeons and 10 residents) from February 2018 to April 2019. Data analysis followed the IPSE analytic procedure and was conducted in two stages: three individual researchers carried out independent work and the group collectively pooled data.

**Results:**

Three axes of experience were identified: (i) the loss of meaning, that is being a doctor, no longer has any meaning in the actual context of public hospitals; (ii) “the tower of Babel”, the impossibility of dialogue with both management and colleagues; and (iii) physicians’ daily interactions: too many conflicts, too much pressure and not enough recognition.

**Conclusion:**

Physicians in this study described being exposed to a deleterious atmosphere, experiencing both emotional abuse and structural violence within the workplace. They considered that such an environment could contribute to the development of burnout. Further research is necessary to assess this hypothesis.

In recent years, doctors’ burnout has become a major public health issue in most Western countries (1), having harmful effects on the healthcare system and on physicians and residents themselves (2). Currently, the COVID-19 pandemic has put a considerable strain on healthcare professionals (3). This pandemic has brought new stressors (4) but has mostly heightened existing challenges that physicians have to face that are directly correlated to increase burnout (5). Indeed, experienced physicians and physicians-in-training are exposed to psychological distress and psychiatric disorders (6, 7). Burnout prevalence among them is quite high in many countries, regardless of the specialty (8). In a 2015 US study, 54.4% of a sample of 6880 physicians had experienced at least one symptom of burnout (9).

There are many issues regarding the current burnout research. Many criticisms are raised about methodological errors related to its measurement, especially against the questionnaire used in 90% of the studies (10): the Maslach Burnout Inventory (MBI) (11). This questionnaire is based on only one definition of burnout: a work-related syndrome involving emotional exhaustion, depersonalization, and a reduced sense of personal accomplishment (12). There is also an ongoing debate about burnout as a diagnostic entity (13): is it a validated diagnosis because of some clinical specificities? Do burnout symptoms overlap with those of depression but have specific triggers? With current definitions in excess of 40, burnout is a very complex phenomenon supported by diverse theories (14–17). More recently, an international expert panel reached a consensual definition, without any theoretical underpinnings, that “occupational” burnout is an “exhaustion due to prolonged exposure to work-related problems” (18). This definition has, however, been criticized for not specifying the nature of work. The term “occupational” suggests that burnout can only occur within the context of formal employment (19).

There is an abundant scientific literature on physicians’ burnout. The aspects most studied are the outcomes of burnout across regions and specialties (20), the individual and organizational factors that contribute to or protect against it (21), and the efficacy of targeted interventions (22). Qualitative studies of physicians’ burnout have developed in recent years. Qualitative methods are especially relevant in this context. They are a tool of choice for focusing on the views of the physicians of how they experience, conceive, and understand burnout in their own field. In 2019, we conducted a systematic review of this literature and identified 33 articles (23). These qualitative studies explored physicians’ burnout contributing and protective factors, mostly at individual and organizational levels. Our analysis of this review also showed an intermediate level of these factors, that is the group and interpersonal relationships within the workplace close environment. This group and interpersonal level is an original axis for innovative protection and intervention for battling doctors’ burnout, and implies the consideration of burnout as an individual experience taking place within both a group and workplace environment.

Socialization at work has been described as being protective (21). Relational and group dimensions within the workplace have been researched in the fields of social psychology and sociology of work (24).

Yet, to our knowledge, no study has ever explored the experience of physicians’ burnout focusing on the group and workplace environment around it.

The aim of this qualitative study was to fill this gap and explore in depth the workplace environment of French hospitals’ departments in which one or several physicians’ burnout occurred. More precisely, we wanted to investigate to which environment, context and work-related problems physicians are exposed in in such departments.

## Method

This exploratory national qualitative study used the inductive process to analyse the structure of lived experience (IPSE) approach (25), a qualitative method specifically developed for clinical medical research to reach concrete proposals. This approach relies on an inductive process exploring in-depth the lived experience of patients, their loved ones and healthcare providers as well as the analysis of the structure of lived experience. Five stages structure the entire research process. The report of this study complies with the COREQ guideline (26). This study was conducted from January 2018 through April 2019 and was approved by the “Comité consultatif de l’Information en matière de recherche dans le domaine de la santé (CCTIRS, ref 15903)”, the “Commission Nationale de l’Informatique et des Libertés (CNIL, ref DR-2016-011) and is registered in ClinicalTrials.gov (NCT02893020). All participants provided informed written consent before inclusion.

### Stage 1: Setting up a research group

Our research group included one male gastrointestinal surgeon (OF), two psychiatrists (one woman/one man), both researchers specialized in qualitative methods (ARL, JS), three female psychologists trained in qualitative methods (LB, EM, CS) and a hospital specialist who herself experienced burnout (LV). The group’s members were highly diverse, especially in their knowledge, age, and backgrounds. The group worked continuously on reflexivity during open discussions between the researchers.

### Stage 2: Ensuring the originality of the study

Two members of the group (JS, ARL) reviewed the qualitative and quantitative literature systematically to confirm the relevance and originality of the study. They verified that no qualitative study had ever explicitly explored the workplace environment in relation to burnout in public hospital. To remain inductive and open to novelty, the other group members had access to this review only after the data analysis had been completed.

### Stage 3: Recruitment and sampling, aiming for exemplarity

The research group defined the inclusion and exclusion criteria (table 1), which was intended to attain exemplarity. Recruitment was aimed at participants who have experienced quintessential or archetypal examples of the situation being studied. We also endeavored to include participants who might add something new to what was previously found.

**Table 1:** Inclusion and exclusion criteria.

Inclusion criteria	Exclusion criteria
Resident or doctor practicing at a public hospital in any of these specialties: ­gastrointestinal surgery, gastroenterology, and radiology	Current lawsuit against the hospital or one member of the hospital
Working in a department in which at least one doctor/resident has presented burnout: - Within the year preceding the interview - Diagnosed by a psychiatrist - Sick leave	Acute psychiatric and/or somatic symptoms hindering the conduct of a research interview
Two sub-groups: - The person who has experienced burnout himself/herself - The colleague of this person	
Agreed to participate in the study	
Fluent in French	

Our position in this research was ecological and not individual. We decided to include both physicians with and without burnout working in the same departments in order to reach an intersubjective description of the lived experience of the shared workplace environment and avoid being confined to the sole perspectives of physicians suffering from burnout.

Radiology, gastroenterology and digestive surgery departments represent three different typical clinical hospital-based activities in terms of both individual practice and group work – inpatient and outpatient work, operating rooms, interventional activity – but have frequent interactions in daily practice and through multidisciplinary meetings. Recruiting physicians within these three types of departments allowed the exploration of various experiences in a homogeneous framework.

Thanks to the networks of the three French professional societies of these specialties, we were able to identify several departments (N=13) in France that had at least one doctor suffering from burnout. To operationalize this criterion, a psychiatrist had to diagnose the burnout within the year preceding in an interview, and the burnout was associated with a related sick leave. In each department, we aimed to interview at least one physician with a lived experience of burnout and at least one of their direct colleagues. In a preliminary interview by telephone or face-to-face, we described the study to participants and verified they met the inclusion criteria. To attain exemplarity, sampling strategy was purposive with maximum variation (27) to select doctors that differed by sex, age, family status, years of experience, rank in their department, and medical practice.

Sample size was not defined in advance but was determined by data saturation according to the principle of “theoretical sufficiency” (28). Inclusion of new participants continued until the analysis of new material no longer yielded new findings; that is, data collection and analysis were complete when the group of research considered that the axes of experience obtained provided a sufficient explanatory framework for the data collected. Saturation is a key criterion for validity in qualitative research as it ensures in-depth study of the phenomenon and suggests that further interviews are unlikely to produce new findings.

### Stage 4: Data collection, access to experience

From February 2018 through April 2019, two researchers (LB, CS) conducted the interviews. They met each participant, obtained his/her written consent and collected social/demographic data to facilitate the subsequent research interview. A few days later in the participant’s workplace, they conducted semi-structured one-on-one interviews using an open-ended approach, structured by areas to explore topics. These areas (table 2) were collectively determined by the group based on the assessment of two pilot interviews. The interviewers used an interactive conversational style. In an IPSE study, participants are considered the experts on their own experience and researchers must conduct interviews that offer them the opportunity to recount it. The interviews lasted 60–90 minutes. They were recorded and transcribed into anonymized verbatim, including the participants’ expressive nuances. These transcripts were then analyzed. Interviewers took field notes after every interview in order to better explore their reflexivity during group meetings.

**Table 2.** Interview guide.

Area of experience	Potential questions
Daily life at work	Can you tell us about a regular day at the hospital?
Workplace environment	Can you describe the environment in which you are working? What is the usual atmosphere?
Burnout	Can you tell us what happened to you/your colleague?
Emotional experiences	How do you feel when you are at work? What kind of emotions?
Interpersonal relationships	Can you tell us about the relationships with your colleagues in your workplace?

### Stage 5: Data analysis, from the description of the structure of experience to practical implications

**Figure 1:**
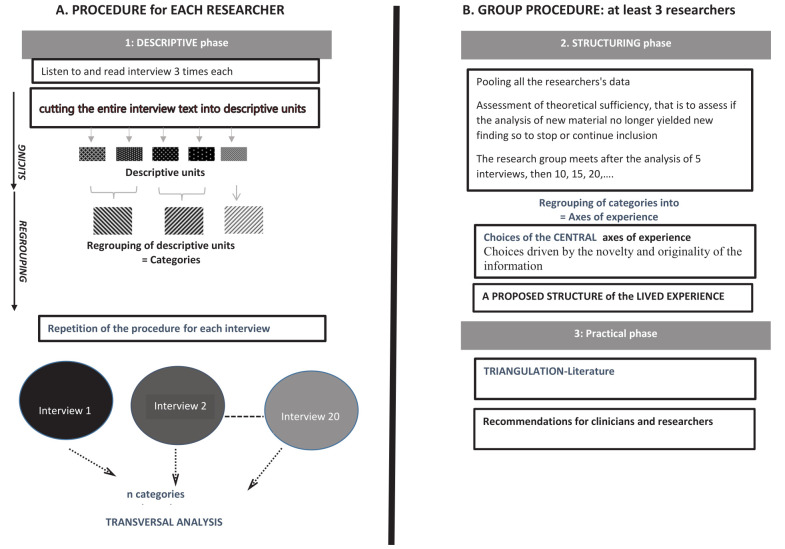
Data analysis procedure

The analytic IPSE process presented in figure 1 has been detailed elsewhere (25). It relied on an inductive, phenomenological method based on two stages: three individual researchers carried out independent work and the group collectively pooled data. The individual procedure consisted of three qualitative researchers (JS, LB, CS) independently and simultaneously conducting a systematic descriptive analysis aimed at conveying each participant’s experience. This involved for each interview: (i) listening to the recorded interview twice and to reading it three times; (ii) exploring the experience word by word, that is cutting up the entire text into descriptive units; (iii) regrouping the descriptive units into categories. These stages are carried out with the help of QSR NVivo 12 software. During the group process, these three researchers and the other group members – familiarized with the data through listening and reading all the interviews as many times as necessary – met nine times, after the analysis of five interviews, for two-hour meetings in order to conduct (i) the structuring phase, that is to regroup the categories into axes of experience; these axes being constructed such that each can be linked to its subjacent categories, and then to determine the structure of lived experience characterized by the central axes; and (ii) the practical phase, a process of triangulation with the data in the literature to identify the original aspects of the results.

### Criteria for rigor in the analyses and patient and public involvement

We used several criteria to ensure the rigor of the analysis and the trustworthiness of the results: triangulation, attention to negative cases, reflexivity within the group process, and feedback from “subjects of the experience” by presenting the research to a group of physicians and residents (N=20) from other medical and surgical departments – dermatology, anatomopathology, internal medicine, reconstructive surgery and psychiatry. They all recognized their own experience in the structure we proposed. This ensured the transferability of our results.

## Results

We included 45 physicians and residents working in 13 departments, including 16 who had experienced burnout and 29 of their colleagues. Every potential participant we reached agreed to participate. For 3 departments, two physicians had experienced burnout. Among the colleagues, some reported being under psychotropic medication or seeing a psychotherapist. More than half of them mentioned feeling at risk of developing a burnout and/or having been “almost in burnout”, or in what many called “a pre-burnout”. The general characteristics of all participants are described in table 3.

**Table 3:** Summary of participants’ characteristics.

Physician characteristics	N (%)
Gender	
Men	26 (58)
Women	19 (42)
Age (mean years)	36
Marital status	
Married, or in a civil union	30 (66)
Living together	5 (11)
Unmarried	10 (22)
Number of children	
0	16 (36)
1	7 (16)
2	11 (24)
3	7 (16)
>3	4 (8)
Physicians diagnosed with burnout	16 (36)
Colleagues	29 (64)
Antidepressant medications	3(6)
Anxiolytics medications use	6(13)
Psychotherapy	4(8)
Specialities	
Radiologists	13 (29)
Gastroenterologists	12 (27)
Gastrointestinal surgeons	10 (22)
Residents	10 (22)
Public Hospitals	
Paris	
8 departments	30 (66)
Dijon	
1 department	2 (4)
Rouen	
2 departments	6 (13)
Grenoble	
2 departments	7 (16)

The data analysis showed that the structure of experience around workplace environment in this particular situation was common for both the physicians who experienced burnout and their colleagues. All participants reported experiencing a specific atmosphere around burnout in their workplace. We identified three central axes, which will be described in detail below, focusing only on negative aspects of the workplace environment: (i) the loss of meaning, (ii) the impossibility of dialogue; and (iii) physicians’ daily interactions: too many conflicts, too much pressure and not enough recognition. The relevant quotations (from the interview transcripts, translated from French into English for the sole purpose of this article) are shown in supplementary material (https://www.sjweh.fi/article/3977) table S1.

### The loss of meaning

According to most of the participants, being a doctor, even a good doctor, no longer has any meaning in the current context of public hospitals [quotation 1 (Q1) in supplementary table S1].

### Caring is no longer the priority

Most of these specialists considered that their primary function as a physician (treating patients, training residents and students) was being diverted to non-medical tasks (administrative and to make up for the lack of supplies and staff: replacing missing orderlies to move patients on stretchers, substituting in various ways for missing material and the paramedical and medical staff who have not been replaced) (Q2; Q3). Many specified that this primary function was no longer appreciated at all, even sometimes cynically mocked by management or heads of department. The meaning of care was now determined by its cost efficiency and no longer by the quality of care: medical knowledge, time spent with the patient, a relationship of trust, or the doctor’s involvement with the patient and the family (Q4; Q5).

### No more passion

The most experienced doctors reproached the new generation saying they practice medicine with neither passion nor devotion or commitment as they did. The residents we interviewed reported these criticisms and explained that, given the current constraints in their profession, they do attribute a greater importance to their personal lives (Q6).

### No freedom and no vision

Participants complained that they no longer have any freedom or free will in practicing their profession (Q7; Q8). They felt that vision and continuity were absent in the organization of their departments. They recounted an endless succession of orders and counter-orders, that led them in one direction and then backwards to deconstruct what they had just finished building (Q9). Accordingly, each new order and each counter-order was perceived as an accusation that their work did not satisfy management and never would. They experienced these contradictory injunctions as an implicit form of abuse. Many considered the lack of free will and constant dissatisfaction of management as “harmful” or “deleterious” for the physicians. Some perceived it as a potential cause for physician’s burnout (Q10).

### The “tower of Babel”: the impossibility of dialogue

*A “dialogue of the deaf” with management*. Most doctors insisted that dialogue with hospital management was impossible. They did not think that management understood them or had any idea at all of their profession and its constraints. They perceived clearly that they did not speak the same language or share the same values: while management talked to them about numbers and cutting costs, they were discussing essential care and serious diseases (Q11). They thought that management has never heard their requests or reports related to important problems (lack of beds, lack of time slots, turnover, lack of resources, etc.) or taken them as seriously as the situation required (Q12; Q13). Not being listened to and understood was also experienced as a form of abuse that could “contribute to burnout” on its own.

*The impossibility of dialogue with colleagues*. The doctors explained they were unable to have conversations with their paramedical colleagues about their difficulties. The other healthcare workers saw them as “privileged”, which impeded the expression of any complaints and the possibility of mutual aid, which was evoked as a memory of a long-ago time now gone (Q14).

Even among doctors, the participants reported that it is extremely complicated to have real dialogue, understand each other and resolve conflicts, especially for doctors of different generations and specialties (Q15).

### Physicians’ daily interactions: too many conflicts, too much pressure and not enough recognition

Finally, all the doctors described daily interactions to lack recognition and be full of conflictual and pressuring interactions among doctors, between doctors and other healthcare workers, between doctors and hospital management, and with patients. Although participants only reported few situations of explicit violence – either physical or verbal – threats of violence and situations close to becoming violent, they mostly used the French term “violence” to describe these daily interactions and made some causal inference between them and physicians’ burnout. Most of the time “violence” was used in a figurative sense, which is much more common in French than English, and was associated with other terms such as “harassment”, “abuse”, “conflicts”, “humiliation”, “submission”, “pressure”, and “perniciousness”. The physicians reported four distinct situations.

*Severe conflicts with management* linked to an inability to control one’s emotions or recurrent conflicts between people or with management (Q16). Some events seem propitious to the externalization of these conflicts (department meetings, division meetings, orders from colleagues, working conditions (Q17).

*Daily horizontal conflicts* directly linked to harassment by a supervisor or colleague. Most of the time, the doctors witnessing or experiencing these situations of harassment blamed the hospital system for promoting individualistic, competitive, callous, or even “*megalomaniac and pernicious”* staff to positions of responsibility (Q18; Q19). Still more serious, some participants considered that medical culture, its hierarchies, its “traditions” and its “omerta” – a term used by several participants referring to an implicit code of silence about conflicts and harassment within hospitals – enable some doctors in high positions to harass other doctors, especially, women and residents. (Q20; Q21).

Female doctors mentioned the pressure they experienced during their pregnancies and maternity leaves; they did not allow themselves to show any signs of fatigue related to their pregnancy. Some reported that they were sometimes ordered to shorten their maternity leave to keep the department running smoothly; they felt guilty toward their colleagues, already understaffed, when using the entire length of their maternity leave. They considered that for women, especially in surgery, becoming pregnant and having children were impediments to professional advancement (Q22). At the same time, young doctors recounted frequent insults and verbal violence (“*young slacker”*) by some of their department heads, comments that humiliated them.

*Constant pressure by management and lack of recognition:* participants reported that management clearly instructed doctors to *“do more with less”*. They also expressed a lack of recognition, regarding both their status (what some called “doctor-bashing”) and their essential role within the hospitals (Q23; Q24). Some doctors even thought of burnout as a method of human resource management: when an individual “cracked”, he or she was replaced by a doctor more submissive to the laws of the new administrative management (Q25).

*“The patients, they changed”*: Many doctors noted changes in their relationships with patients, who were described as more “demanding” more “aggressive” and “less grateful” to doctors than in the past (Q26; Q27). Some physicians also talked about being sued or prosecuted and reported threats of violence from patients (Q28).

## Discussion

Among the colleagues of physicians with a burnout experience, more than half reported also being in distress. It was not intentional to recruit this proportion of colleagues in distress. However, it is consistent with epidemiologic data, for instance in the study of Shanafelt et al (9) more than half of the sample of 6880 physicians reported at least one symptom of burnout. The current social and economic context of French hospitals – as it is in many European countries – could also explain this proportion of distress among the direct colleagues. Nowadays, in order to avoid closure, hospitals must adopt a profit-making view. A German qualitative study has described how this economic pressure on hospitals could impact medical practice and lead to stressful situations and personal frustration among doctors (29). Moreover, organizational burnout contributors are more likely to be present in these departments in which at least one case of burnout occurred, and this might also be another explaining factor.

The risk of burnout among healthcare workers has been mostly associated with the emotional burden of their work (30) and the lack of human/material resources in hospital departments (31). However, burnout has been described as a changeable concept: its exact meaning varies with its context and the intentions of those using the term (32). In this study, all the participants, both doctors who experienced burnout and those who did not, mentioned environmental factors that have been already described in the literature as burnout contributors (33, 34). There were either organizational – paperwork load, the constant need to do things faster, the hospital chain of command, and the pressure of economics, cost-cutting, and numbers to be achieved (33) – or interpersonal such as discrimination, relationship problems in the team, and lack of recognition (34).

The first original aspect of our results is the common description, in these departments, of a deleterious atmosphere. Physicians, both with and without burnout or even psychological distress, were exposed to this deleterious ambience they characterized by an absence of meaning and recognition of their medical work, the impossibility of dialogue with management and between themselves and negative daily interactions such as pressure, harassment, abuse, conflicts or even violence. The overuse of the French word “violence”, in all the narratives and mostly in a figurative sense, raises the question whether we should consider these aspects as part of workplace violence. The association between physicians’ psychological distress in general, burnout in particular, and workplace violence has already been shown in several quantitative studies (35, 36). Some studies focused mostly on the violent behaviors by patients toward doctors and have pointed out the increase in workplace violence and its harmful effect on care (37). Others described *horizontal violence* between doctors (38), the best documented example in our study being bullying of female doctors and residents by other physicians.

The definition of workplace violence is quite restrictive, that is “incidents where staff were abused, threatened or assaulted in circumstances related to their work, involving an explicit or implicit threat to their safety, well-being or health” (39). It does not consider features such as lack of dialogue, meaning or recognition but also pressure, harassment, or constant conflictual interactions. Yet, all these aspects were experienced as insidious abusive acts or mistreatments by the participants of our study. We think that this deleterious ambiance could relate more with both structural violence and emotional abuse, that is forms of violence that are non-physical and sometimes non-intentional. Structural violence occurs when a social institution – here the French public hospital – may harm people by preventing them from meeting their basic needs (40). In our results, these basic needs could be working with meaning, dialogue, and recognition. Structural violence has already been described within the hospital workplace (41), but to our knowledge no research has ever addressed any direct association between structural violence and physicians’ burnout. Emotional abuse is characterized by persistent, repetitive patterns of verbal and nonverbal – but nonphysical – behaviors that harm or intend to harm the targeted person (42). This form of abuse has already been reported by physicians who suffer from burnout, especially residents and women (43).

The second original aspect of our results is that participants perceived and/or experienced this deleterious workplace ambience as potentially causing burnout. Further research is necessary to confirm whether this correlation perceived by the participants is a valid hypothesis or not. Given the fact that our results do not distinguish between physicians who experienced burnout and those who did not, they could serve as a relevant support to elaborate a quantitative study to test this hypothesis by screening all the aspects of this deleterious ambience with both groups. Such an approach could help determine which aspects are the most salient and significatively correlated with physicians’ burnout, so they could be targeted as a priority. Moreover, since physicians without burnout or even psychological distress also described being exposed to such a deleterious atmosphere, if a correlation is found, it would be particularly relevant to fully describe the coping strategies and protective factors used by those physicians in order to draw concrete preventive implications.

Even if this study was conducted before the COVID-19 pandemic, we believe that concrete implications drawn from our results can be already transposed to this context. Concrete actions to help physicians with work-related psychological distress in this distinctive time, within departments in which burnout occurs, would be to directly intervene in the workplace by: (i) allowing physicians to focus mainly on medical tasks and relieving them of tasks less essential for care; (ii) promoting the essential role physicians play within the healthcare system; (iii) increasing awareness of workplace bullying, harassment and abuse especially targeted at residents and female doctors; and (iv) facilitating dialogue and solidarity among healthcare professionals and between doctors and management.

### Study limitations

First, this study took place in France. Caution is needed when transposing our results to other places, especially non-Western countries, because the public hospital context depends strongly on the organization of the medical system as well as on the country’s economy. Second, our results were common to all the doctors. Subgroup data analyses did not show any differences between either the specialties, the age or gender of participant. Further qualitative studies should in-depth explore the lived experience of residents and female doctors. Indeed, both appeared to be more exposed to the deleterious environment described in our results.

Third, our sample focuses only on physicians’ perspectives. Future studies could explore the perspectives of paramedics and other non-doctors’ colleagues about the workplace environment related to physician’s burnout in similar or the same departments to identify similarities and differences.

Finally, in the context of recent burnout, participants focused on negative aspects of interpersonal relationships and workplace environment. Data analysis of the interviews revealed that positive aspects were not even a minor theme. No “negative cases”, ie, cases that would differ from this structure of lived experience and reporting for instance positive aspects, were found among the 45 participants. This focus on negative aspects might result, we think, from two factors. First, the interview might have been seen as an opportunity to complain. Second, the potential inhibition or reluctance of physicians to speak about positive aspects could also be out of loyalty and solidarity with their colleagues with burnout. A study with similar design within departments free of physician’s burnout should be conducted to explore and describe protective factors related to the workplace environment.

### Concluding remarks

Physicians in this study, whether they had experienced burnout or not, described being exposed to a deleterious atmosphere, close to both emotional abuse and structural violence within the workplace. They considered that such an atmosphere could contribute to the development of burnout. Further quantitative research using the findings of this study could confirm such correlations and enable the drawing of concrete preventive implications.

### Financial material support

This study was funded by a grant from the French Ministry of Health (PREPS 1400591N) awarded to OF. The funder did not have any role either in the study design, in the collection, analysis, and interpretation of data, or in the decision to submit the article for publication. Researchers remained independent from the funder.

### Conflict of interest

All authors have completed the ICMJE uniform disclosure form at www.icmje.org/coi_disclosure.pdf and declare: no support from any organization for the submitted work; no financial relationships with any organizations that might have an interest in the submitted work in the previous three years; no other relationships or activities that could appear to have influenced the submitted work.

### Protection of research participants

The study was approved by the “Comité consultatif de l’Information en matière de recherche dans le domaine de la santé (CCTIRS, ref 15903)”, the “Commission Nationale de l’Informatique et des Libertés (CNIL, ref DR-2016-011) and is registered in ClinicalTrials.gov (NCT02893020). All participants provided informed written consent before inclusion.

## Supplementary material

Supplementary material
