# Supplementary material for: Workplace environment around physicians’ burnout: A qualitative study in French hospitals
Source: Scand J Work Environ Health. 2021 Sep 30;47(7):521–30. doi: 10.5271/sjweh.3977 (PMC8504159; doi:10.5271/sjweh.3977)
Supplement: Supplementary material [file SJWEH-47-521-S001.pdf]

Workplace environment around physicians' burnout: A qualitative study in French hospitals[1]

by Jordan Sibeoni, PhD,[2] Laura Bellon-Champel, PhD, Laurence Verneuil, PhD, Caroline Siaugues, PhD, Anne Revah-Levy, PhD, Olivier Farges, PhD

1. *Supplementary table*
2. *Correspondence to: Dr Jordan Sibeoni, Service Universitaire de Psychiatrie de l'Adolescent, Argenteuil Hospital Centre, 69 Rue du Lieutenant Colonel Prud'hon, 95107 ARGENTEUIL Cedex, France. [E-mail: jordansib@hotmail.com]*

Table S1: quotations

**1. Loss of meaning**	
1.1 Caring is no longer the priority	P24-Q1: They fall, because there's a moment when they no longer understand what they are doing, order, counter-order. There's no more sense.<br>P13-Q2: Two nurses are necessary, there's one nurse missing ... it's the lack of resources, or their poor distribution that makes the profession impossible…<br>P15-Q3: bad organisation, disorganisation, the hospital is not adapted. Activity increases 10% a year, and we have fewer nurses and fewer secretaries. So, automatically, there are more things that we're responsible for. I do my mail, I don't have a secretary…so I make mistakes automatically. We have fewer nurses, fewer surgical beds than before, while we have more surgical work.<br>P4-Q4: Before, 80% of visits took place in the patient's room. Today 20% are in the room and 80% in front of a computer screen.
1.2 No more passion	P9-Q5: You always have to fight to show what you're doing…you do work that loses money, while they ask us to save money, which is not always obvious in a university hospital centre when you are dealing with diseases [where cost-cutting is not necessarily possible]…It's sometimes difficult to have the impression of fighting for your patients, and then they tell you that you don't bring in any money and therefore you're not interesting. That's hard.<br>P26-Q6: the old generation, they have the impression that they're real tough guys … to use the expression of an old professor, we're just slackers who think only about having fun …, there are a lot of us, however, who'd like to also have a life.
1.3 No freedom and no vision	P14-Q7: burnout can be explained, because doctors no longer find medicine satisfying. For the department head, the problem, it's that management is omnipresent, the pressures … about cutting costs, closing beds, the discussions with management where they have no idea what we do, the contradictory directives....They are always asking us to do more with always fewer resources.<br>P 40-Q8: Now we only deal with accountants who look at their balances at the end of the month...that's all that matters. Medicine, patients and all that, results, survival, complications, they don't care! It's money that counts, only that!<br>P28-Q9: We always have directives that contradict each other. The first year she (the director) arrived, we had to do everything on an outpatient basis. So, we increased our outpatient activity enormously. And then the next year, she told us that there was no longer enough traditional hospitalisation, so we had to increase the number of in-patients…<br>P16-Q10: Everything was completely contradictory. As a result, it was unmanageable … it was exhausting, actually. We never do things right ... We never do things right. (…) If you think about it, no wonder why some physicians lost it, I mean they tell you what you have to do, you do it, then they change their minds and they ask you to do it again but differently, so you do it but still, it won't be good enough (…) how exactly the crazy circle can end? Well Burnout!

**2. The tower of Babel: the impossibility of a dialogue**	
2.1 A dialogue of the deaf with management	P3-Q11: There is a kind of non-dialogue with the administration…a dialogue of the deaf…with different forms of reasoning…<br>P 17-Q12: It's the organisation that is really a problem, there's a patient…there's no bed in the hospital…and this woman…she's been there for 24 hours, in the emergency department. There are people who explain to you that they are in the middle of having emergency crisis meetings, but there are beds in the hospital. And…I find that unacceptable, that infuriates me…it makes me sick, it…the general 'I don't care' attitude.'<br>P19-Q13: When we talk to her [the director] about our problems about nurses, who can't take it anymore in the department, with the turnover, because they're exhausted, that's not the problem for her. When she wants us to close beds, and we tell her that it's already unmanageable to find beds for all our patients, she doesn't hear it…really, we have the impression we're all alone, battling windmills, and then swimming backwards...
2.2 The impossibility of dialogue between colleagues	P31-Q14: when I was younger, the nurses, they had some empathy…they used to say to us: 'you didn't sleep, I'll make you a coffee…it'll be ok, you can do it… You could cry for five minutes, you could say it's hard. She'd say, 'yes, I know it's hard…' And then it was off again. Now, you go see the young nurses, she looks at you, and she goes: 'Mmm, you chose it!' OK, I won't say anything anymore.<br>P22- Q15: It's fine to scream at me for something stupid I did.… But, on the other hand...if it's not my fault, and this has already happened to me…one of the department heads, we were yelling at each other in the hallway because I knew it wasn't me who'd been stupid, and we were screaming at each other.
**3. Physicians' daily interactions: too many conflicts, too much pressure and not enough recognition**	
3.1 Severe conflicts with management	P42-Q16 There was a [hospital] manager there...to tell you how much they mistreat us….In front of the full waiting room...she says to me, 'you do your work badly.'…She's not my superior, she's administration… I say to her: 'Excuse me?' 'Yes, we've checked your file…' …I tell her: 'that would astonish me, because I think I'm the only person who touches it'…So I blew up, I insulted her…<br>P24- Q17[about an administrative director] When I see a "bastard" who treats his heads of departments like sub-shit, insulting them, this is management that I don't appreciate.…The guy turns toward me, I say 'watch out, you're going to get it.' Normally…it shouldn't start with, 'watch out, you're going to get it' because it means I'm getting intimate and insulting. I have no more respect at all [for them].
3.2 Daily horizontal conflicts	P10- Q18: There are a lot of people who say we have a difficult profession… I think there are…bosses who persecute us.<br>P14-Q19: Some team have harassers… and they're really a source of burnout.…there are surgeons who are specialists in always undermining the others, or undermining the residents… there was a professor who harassed … he was constantly running us down … he laughed at us, said we were worthless.…It added to our discomfort, it was a daily pressure, and we were at the point where we ended up anxious every time we operated … we were being humiliated, in front of the patients. It was permanent daily harassment.<br>P12-Q20: The residents were treated like dogs. The department head called all the shots: 'I don't want to see you in the OR, I don't want you to go see a patient, you stay in the office.' He stayed in an office all day doing nothing. So, with a treatment…very very…psychologically very hard…It was done deliberately…a department head, moreover, who lost his job after a resident threatened to kill himself.<br>P13- Q21: the concepts of machismo in surgery, there are a lot of women who…who said they didn't feel they were treated as equals. They felt in any case that there was inequality uh...that's related to sex in their specialty.<br>P14-Q22 And in our surgical culture, you never say that it's not ok. Especially you can never say it's not going well, when you're a girl…That's advice they gave me at the beginning of my residency…I was told, I know it's difficult [your pregnancy], I know you're not feeling well, but never say so.

3.3 Constant pressure by management and lack of recognition	P20-Q23: It's organisational, and then the lack of recognition. We take on lots of responsibilities, and it's no longer recognised. Doctors have to be put back at the centre of the process; we are, after all, the chemistry that makes the hospital work. We bring the patients, the techniques, take on the responsibilities. We're no longer at the centre. I think that's the big problem. Before, at the hospital, we felt nonetheless that our investment was recognised.<br>P6-Q24: towards all doctors, there's been a kind of doctor-bashing for years now…an absence of recognition.<br>P24-Q25: We have, after all, a director who's had three trials for psychological harassment, in court, criminal cases.…they do everything to make people burn out. Because when the person burns out, you can replace him by someone else. It's the new way to fire people, a new way to manage human resources. What I'm telling you is terrible, but I think it terrible.<br>P19-Q26: You can have people a little aggressive…consultations like that, it can be kind of heavy, yeah...on your mood at the end of the day.
3.4 "The patients, they changed"	P2-Q27: a complete absence of recognition, even… substantial aggression uh…by patients who consider that medicine is…something that they're owed, just like you go to the post office or when you go…and that, it's true…I think that's also a part of the current problem.<br>P41-Q28: When you're on trial, I think that is when you're in a real conflict with some patients, it can be trying.
